# Evaluation of the nonsteroidal anti-inflammatory drug-sparing effect of etanercept in axial spondyloarthritis: results of the multicenter, randomized, double-blind, placebo-controlled SPARSE study

**DOI:** 10.1186/s13075-014-0481-5

**Published:** 2014-11-27

**Authors:** Maxime Dougados, Emily Wood, Bernard Combe, Thierry Schaeverbeke, Corinne Miceli-Richard, Francis Berenbaum, Nandan Koppiker, Arnaud Dubanchet, Isabelle Logeart

**Affiliations:** Paris Descartes University, Department of Rheumatology, Hôpital Cochin, Assistance Publique – Hôpitaux de Paris – INSERM (U1153), Clinical Epidemiology and Biostatistics, PRES Sorbonne Paris-Cité, Paris, France; Rheumatology B Department, Cochin Hospital, 27 Rue du Faubourg Saint-Jacques, Paris, 75014 France; Statistical Consultancy, Quanticate, UK; Department of Rheumatology, Lapeyronie Hospital, University of Montpellier 1, Montpellier, France; Rheumatology Department, Centre Hospitalier Universitaire de Bordeaux, Bordeaux, France; Hôpital de Bicêtre, Paris-Sud University, Le Kremlin-Bicêtre, Paris, France; INSERM UMRS-938, Université Paris 06, DHU i2B, AP-HP, Saint-Antoine Hospital, Rheumatology, Paris, France; Pfizer PGRD, Ramsgate Road, Sandwich, Kent CT13 9N J UK; Pfizer SAS, 23-25 Ave. dur Dr. Lannelongue, Paris, F-75668 Cedex14 France; Pfizer PIO, 23-25 Ave. dur Dr. Lannelongue, Paris, F-75668 Cedex14 France

## Abstract

**Introduction:**

In clinical practice, nonsteroidal anti-inflammatory drugs (NSAIDs) are commonly discontinued after response to biologic therapy is achieved in patients with axial spondyloarthritis (axSpA), but the impact of NSAID discontinuation has not been assessed in prospective controlled trials. The aim of the SPARSE study was to evaluate the effects of the anti-tumor necrosis factor agent etanercept on NSAID intake and conventional clinical outcomes in axSpA patients.

**Methods:**

In the double-blind, placebo-controlled period, patients with active (mini Bath Ankylosing Spondylitis Disease Activity Index (BASDAI) ≥4) axSpA despite optimal NSAID intake were randomized to receive etanercept 50 mg or placebo once weekly for 8 weeks. All patients were advised to taper/discontinue their NSAID intake during the treatment period. NSAID intake was self-reported by diary and Assessment of SpondyloArthritis International Society (ASAS)-NSAID scores calculated based on ASAS recommendations. The primary endpoint was change from baseline to week 8 in ASAS-NSAID score (analysis of covariance).

**Results:**

In 90 randomized patients at baseline, mean age (standard deviation) was 38.9 (11.8) years; disease duration, 5.7 (8.1) years; 59/90 (66%) were human leukocyte antigen-B27 positive; 51/90 (57%) had radiographic sacroiliitis; and 45/90 (50%) were magnetic resonance imaging sacroiliitis-positive. Mean ASAS-NSAID scores were similar between etanercept and placebo groups at baseline (98.2 (39.0) versus 93.0 (23.4)), as were BASDAI (6.0 (1.7) versus 5.9 (1.5)), and Bath Ankylosing Spondylitis Functional Index (5.2 (2.1) versus 5.1 (2.2)). Mean changes (SE) in ASAS-NSAID score from baseline to week 8 were –63.9 (6.1) and –36.6 (5.9) in the etanercept and placebo groups (between-group difference, –27.3; *P* = 0.002). Significantly higher proportions of patients receiving etanercept versus placebo had an ASAS-NSAID score <10 (46% versus 17%; *P* = 0.008) and ASAS-NSAID score of 0 (41% versus 14%; *P* = 0.013) at this time point. Significantly more patients in the etanercept versus placebo group achieved BASDAI50 (39% versus 18%; *P* = 0.032) and ASAS40 (44% versus 21%; *P* = 0.028) at week 8.

**Conclusions:**

In patients with axSpA, etanercept was associated with clinically relevant NSAID-sparing effects in addition to significant improvements in conventional clinical outcomes.

**Trial registration:**

ClinicalTrials.gov NCT01298531. Registered 16 February 2011.

**Electronic supplementary material:**

The online version of this article (doi:10.1186/s13075-014-0481-5) contains supplementary material, which is available to authorized users.

## Introduction

Spondyloarthritis (SpA) encompasses a cluster of rheumatic conditions, characterized by inflammation of the spine, entheses, and peripheral joints, that share an association with the major histocompatibility complex class 1 antigen (human leukocyte antigen-B27) and with clinical extra-articular manifestations, such as inflammatory bowel disease, psoriasis, and uveitis [1]. Classification criteria of the Assessment of SpondyloArthritis International Society (ASAS) distinguish between predominantly axial and peripheral disease manifestations. Patients with back pain persisting for longer than 3 months and symptom onset before 45 years of age are classified as having axial SpA (axSpA) if they have evidence of sacroiliitis on imaging (that is, structural damage observed on plain X-ray images or inflammatory lesions observed on magnetic resonance imaging) in addition to at least one SpA feature (satisfying criteria for the imaging arm) or, in the absence of imaging evidence of sacroiliitis, if they have human leukocyte antigen-B27 positivity and at least two SpA features (satisfying criteria for the clinical arm) [2]. Patients with axSpA on imaging and nonradiographic axSpA have shown similar burden of illness, with comparable levels of disease activity and pain, as well as functional and quality-of-life impairment [3-5].

Since the beginning of the new millennium, the introduction of biologic agents for use in persistent disease has transformed the SpA treatment paradigm. Despite these important developments, nonsteroidal anti-inflammatory drugs (NSAIDs) continue to serve as first-line pharmacotherapy, particularly for axSpA [6,7]. In fact, a good response to NSAID therapy is one of the SpA features included in candidate criteria for both the imaging and clinical arms of the ASAS axSpA classification [2]. NSAIDs effectively reduce pain and stiffness in patients with SpA after 2 to 3 days [8-10] and also may reduce levels of biological inflammatory markers [11]. In addition, some data suggest that NSAIDs reduce progression of structural damage [12-14]. However, the symptomatic, anti-inflammatory, and potential structural benefits of NSAIDs are dependent on their continuous daily use, which may be problematic because of gastrointestinal, cardiovascular, and renal toxicity associated with protracted therapy [15-19]. In light of safety concerns, national health agencies have recommended use of NSAIDs at the minimum effective dose for the shortest possible period [20,21].

Anti–tumor necrosis factor (TNF) agents have been shown to improve signs and symptoms in patients with still-active axSpA despite stable background NSAID therapy in controlled clinical trials [22-28]. In patients who respond to anti-TNF therapy, clinicians may advise continuation of systematic daily NSAID intake in combination with the biologic therapy because of the potential structural effects of NSAIDs and potential lack of structural effects of anti-TNF agents, with the aim of reducing long-term disability. A preliminary study suggests potential structural benefits of anti-TNF agents [29], but these observations need to be confirmed in additional clinical trials. Alternatively, patients may be advised to discontinue NSAIDs once symptoms improve or disappear with anti-TNF therapy to avoid the possible complications of long-term NSAID intake. For many clinicians, the putative structural benefits of NSAIDs do not outweigh the risk of adverse effects.

Although NSAID discontinuation after biologic response in axSpA patients may be common in the clinical practice setting [30], the impact of NSAID reduction or withdrawal has never been evaluated in a prospective randomized placebo-controlled trial in this population. The amount of NSAID intake has been proposed by the ASAS to be a clinically relevant outcome measure for clinical studies in axSpA to evaluate NSAID toxicity as well as the potential NSAID-sparing effects of other treatments [31]. The ASAS-NSAID scoring system has recently been developed as a standardized method of evaluating NSAID intake in clinical trials [31]. The aim of the present randomized, controlled SPARSE study [ClinicalTrials.gov:NCT01298531] was twofold: first, to quantify the effects of treatment with the anti-TNF agent etanercept on NSAID intake in patients with axSpA using the ASAS-NSAID scoring method; and second, to evaluate the safety and efficacy of etanercept in improving the signs and symptoms of the disease.

## Methods

### Study design and study drug

The 8-week, randomized, double-blind, placebo-controlled period of this two-period, multicenter, phase 4, prospective study commenced in May 2011; the study was completed in April 2013. All patients were enrolled at and the study conducted in 19 centers in France. At the screening visit, investigators requested that patients discontinue their NSAID and restart the NSAID only if they experienced symptom flare, adjusting treatment as needed to provide optimal symptomatic control. Patients who remained asymptomatic without NSAID treatment during the 2-week to 6-week screening period were considered ineligible and were withdrawn from the study. Patients who experienced a flare of symptoms after discontinuing their NSAID and had restarted NSAID treatment were randomized using an interactive voice response system (Impala NY, NY, US) in a 1:1 ratio to receive either etanercept 50 mg or placebo subcutaneously once weekly for 8 weeks, in addition to their background NSAID. All patients were advised to taper/discontinue their NSAID intake during the study treatment period.

Patients and physicians remained blinded to treatment assignment throughout the 8-week study period. Patients randomized to either treatment group were permitted an early escape to open-label etanercept 50 mg once weekly at week 4 if they experienced >50% increase from baseline in total back pain or the Bath Ankylosing Spondylitis Disease Activity Index (BASDAI) [32] despite receiving NSAIDs at the maximum tolerated dosage. All patients who completed the double-blind period were eligible to receive etanercept 50 mg once weekly plus background NSAID during a subsequent 8-week open-label treatment period.

The study was conducted in accordance with the International Conference on Harmonization guidelines for good clinical practice and the Declaration of Helsinki. Study activities were not initiated until patients provided informed consent. The study was approved by the central independent review board of the Comité de Protection des Personnes Ile de France VIII, Hôpital Ambroise Paré 9, Avenue Charles de Gaulle, Boulogne Billancourt 92100, France (Chairperson: Dr Frédérique Barthod).

### Inclusion/exclusion criteria

Adult patients were eligible for the study if they had axSpA, as defined by ASAS classification criteria [2]. Active axial involvement was required, defined by mini-BASDAI [33] ((Question 1 + Question 2 + (Question 5 + Question 6) / 2) / 3 ≥ 4 at screening and study baseline), with an inadequate response to at least two NSAIDs taken at the maximum tolerated doses (determined from medical history) for a total combined duration of more than 1 month. Enrolled patients were required to have received an NSAID for at least 5 days per week at two-thirds the maximum licensed dosage for 4 weeks before screening and 1 week before baseline. Patients were ineligible if they received previous biologic treatment; >10 mg/day prednisone or equivalent (or changed dose) within 4 weeks of baseline; or an intra-articular, intravenous, intramuscular, or subcutaneous corticosteroid within 6 weeks of screening. They were also excluded if they had uncontrolled inflammatory bowel disease or uveitis.

### Outcome measures

Study data were collected in compliance with ASAS recommendations for clinical trials of SpA [34]. Imaging was read locally by the radiologist or rheumatologist providing care for the patient. The primary endpoint was the change from baseline to week 8 in the ASAS-NSAID score [31], calculated based on NSAID intake recorded in patient diaries. The ASAS-NSAID score takes into account the type of NSAID, the total daily dose, and number of days with intake during the period of interest (that is, 7 days before the respective visit).

A secondary measure of NSAID-sparing effects was the change in ASAS-NSAID score over time. Secondary clinical endpoints included the proportions of patients who achieved ASAS partial remission [35], the BASDAI50 response [32], ASAS20 and ASAS40 responses [36], and patient acceptable symptom state (PASS) during the double-blind and open-label periods [37,38]. Mean scores over time for the BASDAI (0 to 10) [32], Ankylosing Spondylitis Disease Activity Score on the basis of C-reactive protein (ASDAS-CRP) [39], Physician Global Assessment of disease activity (0 to 10), total back pain, Bath Ankylosing Spondylitis Functional Index (0 to 10) [40], and Bath Ankylosing Spondylitis Metrology Index (0 to 10) were also measured. *Post hoc* analyses were also conducted for the proportions of patients achieving other NSAID-sparing endpoints at week 8 (that is, 50% decrease in ASAS-NSAID score compared with baseline, ASAS-NSAID score <10, and ASAS-NSAID score = 0); ASDAS-CRP inactive disease or moderate, high, or very high disease activity levels; and normal levels of C-reactive protein (that is, ≤1.25 × upper limit of normal (4.9 mg/l)) at week 8. Statistical analysis was not performed for the latter two *post hoc* analyses.

### Sample size

The sample size was determined based on the following assumptions for the primary endpoint: a mean ASAS-NSAID score of 100 in both groups at baseline, and mean scores of 50 and 80 in the etanercept/etanercept and placebo/etanercept groups, respectively, at week 8. A target sample size of 39 patients per treatment group was estimated to provide a between-group difference of 30 for change from baseline to week 8 in the ASAS-NSAID score, assuming a standard deviation of 40 and based on at least 90% statistical power and two-sided testing at α = 0.05.

### Collected NSAID diary data

The ASAS-NSAID score was calculated based on NSAID usage completed on diary cards. Patients were requested to record details of NSAID intake for every day of NSAID usage, including the NSAID name, the dose, and number of tablets taken each day.

### Statistical analyses

Continuous baseline demographic and disease characteristic variables were summarized using descriptive statistics in the intent-to-treat (ITT) population, which comprised all randomized patients who received at least one dose of study drug. NSAID-sparing and clinical efficacy and safety analyses were also conducted in the ITT population unless otherwise noted.

The primary endpoint was the change from baseline to week 8 in the ASAS-NSAID score in the ITT population. The ASAS-NSAID score was calculated from NSAID usage completed on the patient diary cards for the previous 7 days for a particular visit. Scores were calculated only if at least 5 of the 7 days were completed. Missing data were imputed based on adjacent data and using the last observation carried forward approach. Analysis of covariance was used for the primary analysis of the primary endpoint, with baseline ASAS-NSAID score and treatment as explanatory variables. No adjustments were made for multiple testing.

The primary analysis of the primary endpoint was repeated in the modified ITT population as a sensitivity analysis; the modified ITT population encompassed all patients in the ITT population, but for ITT patients who entered the escape arm, only data collected for time points up until initiation of open-label treatment were used. An additional sensitivity analysis was conducted with Wilcoxon rank-sum tests stratified by baseline ASAS-NSAID score. Hodges–Lehmann confidence intervals (CIs) were calculated for the treatment difference corresponding to unstratified Wilcoxon rank-sum tests. In addition, a *post hoc* sensitivity analysis was performed using a different approach to missing data imputation. Specifically, when data were missing for a particular day in the diary, the missing data were counted as no intake; both a last observation carried forward approach and a baseline observation carried forward approach (when no postbaseline diary data were available) were used. Analysis of covariance was used in the same manner as in the primary analysis described above.

The conventional clinical response outcomes at week 8 (that is, partial remission, BASDAI50, ASAS20 and ASAS40 responses, and PASS) were analyzed using a logistic regression model, including treatment and the corresponding baseline scores as covariates. The last observation carried forward approach was used for all clinical response outcomes except PASS, for which observed cases were analyzed. These clinical responses were also summarized at weeks 4, 8, 12, and 16 of the double-blind and open-label periods using observed cases. Changes from baseline in continuous NSAID-sparing and clinical endpoints were analyzed using analysis of covariance with treatment and the corresponding baseline score as covariates. The NSAID intake endpoints (that is, 50% decrease in ASAS-NSAID score compared with baseline, ASAS-NSAID score <10, and ASAS-NSAID score = 0) were analyzed using a logistic regression model including treatment and the baseline ASAS-NSAID score as covariates. Missing data were imputed as outlined for the primary endpoint. All statistical testing was two-sided and conducted at the 5% level; CIs were two-sided 95% CIs.

## Results

### Patients

**Figure 1:**
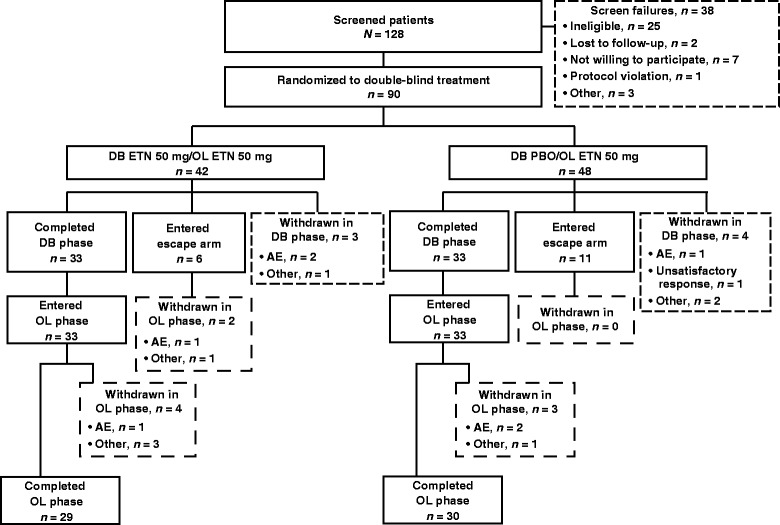
**Patient flow through the double-blind and open-label phases.** AE, adverse event; DB, double-blind; ETN, etanercept; OL, open-label; PBO, placebo.

Of 128 screened patients, 90 patients (etanercept group, *n* = 42; placebo group, *n* = 48) were randomized into the 8-week double-blind treatment period and included in the ITT, modified ITT, and safety populations (Figure 1). Eight patients (19%) in the etanercept group and 10 patients (21%) in the placebo group violated the NSAID inclusion criteria (that is, NSAID intake for at least 5 days/week at two-thirds the maximum licensed dosage during the week before the baseline visit). Fewer patients in the etanercept group escaped early during the double-blind period than in the placebo group (etanercept group, *n* = 6 (14%); placebo group, *n* = 11 (23%)). Eighty-one patients (etanercept group, *n* = 39; placebo group, *n* = 42) were included in the primary analysis of the primary endpoint. At baseline, one patient (2%) in the placebo group reported a missing NSAID diary; four patients (10%) in the etanercept group and 11 patients (23%) in the placebo group had some missing diary information. (A summary of missing NSAID diary data at baseline and during the double-blind period is provided in Additional file 1). A total of 66 patients (etanercept group, *n* = 33; placebo group, *n* = 33) completed the double-blind period and entered the open-label treatment period.

Demographic and disease characteristics at baseline were similar between the treatment groups (Table 1). Fifty-nine of 90 patients (66%) were human leukocyte antigen-B27–positive, 51/90 patients (57%) had radiographic sacroiliitis based on the modified New York criteria [41], and 45/90 patients (50%) had sacroiliac joint inflammation on magnetic resonance imaging based on the Outcome Measures in Rheumatology definition [42]. At baseline, patients had a moderate to high level of disease activity and functional impairment as measured by the BASDAI, ASDAS, and Bath Ankylosing Spondylitis Functional Index. A relatively low level of spinal mobility impairment was suggested by the low baseline Bath Ankylosing Spondylitis Metrology Index.

**Table 1:** **Baseline demographics and disease characteristics**

	**Etanercept 50 mg/etanercept 50 mg (** ***n*** ** = 42)**	**Placebo/etanercept 50 mg (** ***n *** **= 48)**
**Baseline patient characteristics**		
Age (years)	38.8 (12.3)	38.9 (11.4)
Female	18 (42.9%)	16 (33.3%)
White	40 (95.2%)	48 (100.0%)
Weight (kg)	73.8 (14.2)	75.4 (15.2)
Body mass index (kg/m^2^)	25.7 (4.8)	25.9 (4.9)
Human leukocyte antigen-B27–positive	28 (66.7%)	31 (64.6%)
**Disease characteristics**		
Duration since diagnosis of ankylosing spondylitis (years)	6.0 (9.0)	5.5 (7.4)
Past history or present symptoms		
Arthritis	11 (26.2%)	18 (37.5%)
Inflammatory back pain	40 (95.2%)	48 (100.0%)
Enthesitis	25 (59.5%)	33 (68.8%)
Dactylitis	4 (9.5%)	8 (16.7%)
Psoriasis	9 (21.4%)	9 (18.8%)
Uveitis	5 (11.9%)	3 (6.3%)
Family history		
Ankylosing spondylitis	9 (21.4%)	4 (8.3%)
Rheumatoid arthritis	2 (4.8%)	3 (6.3%)
Inflammatory bowel disease	2 (4.8%)	1 (2.1%)
Uveitis	2 (4.8%)	0 (0%)
Positive pelvic X-ray	24 (57.1%)	27 (56.3%)
MRI sacroiliitis positive	21 (50.0%)	24 (50.0%)
**NSAID intake**		
ASAS-NSAID score^a^	98.2 (39.0)	93.0 (23.4)
**Disease activity**		
BASDAI (0 to 100)	6.0 (1.6)	5.9 (1.5)
ASDAS-CRP	3.4 (0.9)	3.2 (0.8)
ASDAS-CRP disease state^b^		
Inactive disease	0 (0%)	0 (0%)
Moderate disease activity	5 (11.9%)	3 (7.0%)
High disease activity	19 (45.2%)	23 (53.5%)
Very high disease activity	18 (42.9%)	17 (39.5%)
PGA of disease activity (0 to 10)	6.4 (1.5)	6.2 (1.3)
Total back pain (0 to 100)	6.7 (1.7)	6.4 (1.8)
BASFI (0 to 100)	5.2 (2.1)	5.1 (2.2)
BASMI (0 to 10)	2.6 (1.8)	2.6 (1.6)
C-reactive protein level (mg/dl)	1.0 (1.3)	0.9 (1.4)
Normal C-reactive protein level^c^	21 (50.0%)	28 (65.1%)

Safety population; data presented as mean (standard deviation) or *n* (%).ASAS, Assessment of SpondyloArthritis International Society; ASDAS-CRP, Ankylosing Spondylitis Disease Activity Score on the basis of C-reactive protein; BASDAI, Bath Ankylosing Spondylitis Disease Activity Index; BASFI, Bath Ankylosing Spondylitis Function Index; BASMI, Bath Ankylosing Spondylitis Metrology Index; MRI, magnetic resonance imaging; NSAID, nonsteroidal anti-inflammatory drug; PGA, Physician Global Assessment. ^a^Last observation carried forward, with imputation, intent-to-treat population. ^b^Inactive disease = ASDAS-CRP <1.3; moderate disease activity = 1.3 ≤ ASDAS-CRP <2.1; high disease activity = 2.1 ≤ ASDAS-CRP <3.5; very high disease activity = ASDAS-CRP ≥3.5. ^c^Normal CRP = ≤1.25 × the upper limit of normal (4.9 mg/l).

### Nonsteroidal anti-inflammatory drug-sparing effects

**Figure 2:**
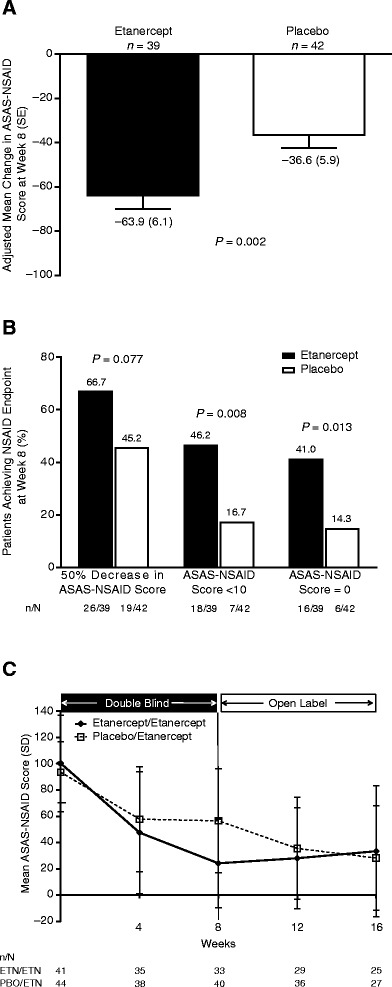
**Nonsteroidal anti-inflammatory drug–sparing effects. (A)** Change in ASAS-NSAID score from baseline to week 8 in patients in the etanercept and placebo groups (primary analysis of primary endpoint). Analysis of covariance, LOCF, with imputation of missing diary data, in the ITT population. **(B)** Proportion of patients in the etanercept and placebo groups achieving other NSAID-sparing endpoints at week 8 of the double-blind period. LOCF, with imputation, ITT population (*n* = number of patients achieving endpoint; *N* = number of patients with analyzable data). **(C)** Mean ASAS-NSAID scores (± SD) in the etanercept/etanercept and placebo/etanercept groups in the double-blind and open-label phases. ASAS-NSAID scores were calculated for observed cases, with no imputation of missing diary data. ASAS-NSAID, Assessment of SpondyloArthritis International Society nonsteroidal anti-inflammatory drug use; ETN, etanercept; ITT, intent-to-treat; LOCF, last observation carried forward; NSAID, nonsteroidal anti-inflammatory drug; PBO, placebo; SD, standard deviation; SE, standard error.

For patients in the ITT population, the mean (standard deviation) ASAS-NSAID score at baseline was similar between the two groups: 98.2 (39.0) and 93.0 (23.4) in the etanercept (*n* = 42) and placebo (*n* = 45) groups, respectively. The primary analysis of the primary endpoint showed a significant difference of –27.3 (95% CI: –44.2 to –10.4; *P* = 0.002) between the etanercept (*n* = 39) and placebo (*n* = 42) groups in the change from baseline in ASAS-NSAID score at week 8 (Figure 2A).

Findings from the sensitivity analyses performed in the modified ITT population (etanercept, *n* = 39; placebo, *n* = 42) and with the Wilcoxon rank-sum test (etanercept, *n* = 39; placebo, *n* = 42) were consistent with those of the primary analysis, with differences of –27.8 (95% CI: –44.8 to –10.8; *P* = 0.002) and –33.3 (Hodges–Lehmann 95% CI: –50.0 to –11.4; *P* = 0.004), respectively. In the *post hoc* analysis of covariance sensitivity analysis of the primary endpoint (etanercept, *n* = 42; placebo, *n* = 47), a similar statistically significant difference of –30.4 (95% CI: –46.2 to –14.7; *P* = 0.0002) was found between the treatment groups.

At week 8, significantly higher proportions of etanercept-treated patients achieved the NSAID-sparing endpoints of ASAS-NSAID score <10 (*P* = 0.008) and ASAS-NSAID score of 0 (*P* = 0.013; Figure 2B). Significant reductions in the ASAS-NSAID score were observed from baseline to week 16 in the etanercept/etanercept group (*n* = 25) and from week 8 to week 16 in the placebo/etanercept group (*n* = 17): –65.93 (95% CI: –87.0 to –44.9; *P* <0.0001) and –39.2 (95% CI: –52.9 to –25.5; *P* <0.0001), respectively (Figure 2C).

### Clinical efficacy

**Figure 3:**
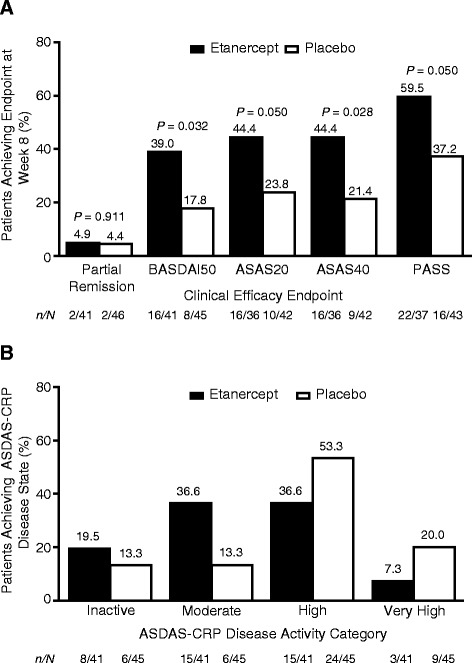
**Clinical efficacy. (A)** Proportion of patients in the etanercept and placebo groups achieving clinical endpoints at week 8 of the double-blind period. Logistic regression, LOCF (except PASS, which was calculated in observed cases). **(B)** Proportion of patients in the etanercept and placebo groups achieving ASDAS-CRP disease activity states at week 8 of the double-blind period. *Post hoc* analysis of ITT population; *n* = number of patients with nonmissing ASDAS-CRP results at each visit. Inactive disease = ASDAS-CRP <1.3; moderate disease activity = 1.3 ≤ ASDAS-CRP <2.1; high disease activity = 2.1 ≤ ASDAS-CRP <3.5; very high disease activity = ASDAS-CRP ≥3.5. ASAS, Assessment of SpondyloArthritis International Society; ASDAS-CRP, Ankylosing Spondylitis Disease Activity Score based on C-reactive protein; BASDAI, Bath Ankylosing Spondylitis Disease Activity Index; ITT, intent-to-treat; LOCF, last observation carried forward; PASS, patient acceptable symptom state.

In the double-blind period, a significantly greater proportion of patients in the etanercept group than in the placebo group achieved BASDAI50 and ASAS40 responses and PASS at week 8 (*P* <0.05; Figure 3A). Numerically greater proportions of patients receiving etanercept achieved all clinical efficacy endpoints compared with patients receiving placebo at weeks 4 and 8 of the double-blind period. At weeks 4 and 8 of the double-blind period, etanercept was associated with significantly greater improvement in most axSpA signs and symptoms compared with placebo, including ASDAS-CRP, Physician Global Assessment of disease activity, total back pain, and Bath Ankylosing Spondylitis Functional Index (Table 2). Although the difference between treatment groups in change from baseline to week 8 in the BASDAI was not statistically significant at week 8 (*P* = 0.051), the difference was significant at week 4 (*P* = 0.015). No significant difference in the Bath Ankylosing Spondylitis Metrology Index was observed between the etanercept and placebo groups at weeks 4 or 8.

**Table 2:** **Absolute changes from baseline (week 0) to weeks 4 and 8 by treatment group (intent-to-treat population, last observation carried forward)**

**Parameter**	**Week 4**			**Week 8**		
	**Etanercept 50 mg**	**Placebo**	***P*** **value**	**Etanercept 50 mg**	**Placebo**	***P*** **value**
	**(** ***n*** **= 39)**	**(** ***n*** **= 44)**		**(** ***n*** **= 41)**	**(** ***n*** **= 45)**	
ASAS-NSAID score	50.3 (6.7)	61.0 (6.5)	0.256	32.4 (6.1)	59.7 (5.9)	0.002
Change from baseline	–46.3 (6.7)	–35.6 (6.5)		–63.9 (6.1)	–36.6 (5.9)	
(95% confidence interval)	(–59.8, –32.9)	(–48.5, –22.7)		(–76.0, –51.8)	(–48.3, –24.9)	
BASDAI (0 to 100)	4.5 (0.3)	5.4 (0.3)	0.015	4.0 (0.3)	4.8 (0.3)	0.051
Change from baseline	–1.5 (0.3)	–0.6 (0.3)		–2.0 (0.3)	–1.1 (0.3)	
(95% confidence interval)	(–2.0, –0.9)	(–1.1, –0.0)		(–2.7, –1.4)	(–1.7, –0.5)	
ASDAS-CRP	2.4 (0.1)	3.1 (0.1)	<0.0001	2.1 (0.1)	2.8 (0.1)	0.001
Change from baseline	–0.9 (0.1)	–0.2 (0.1)		–1.2 (0.1)	–0.5 (0.1)	
(95% confidence interval)	(–1.2, –0.7)	(–0.4, 0.1)		(–1.5, –0.9)	(–0.8, –0.2)	
PGA of disease activity (0 to 10)	4.2 (0.3)	5.6 (0.3)	0.002	3.6 (0.4)	4.7 (0.3)	0.023
Change from baseline	–2.0 (0.3)	–0.7 (0.3)		–2.7 (0.3)	–1.6 (0.3)	
(95% confidence interval)	(–2.6, –1.5)	(–1.3, –0.1)		(–3.4, –2.0)	(–2.2, –0.9)	
Total back pain (0 to 100 mm)	5.0 (0.4)	6.0 (0.3)	0.047	4.3 (0.4)	5.6 (0.4)	0.021
Change from baseline	–1.6 (0.4)	–0.6 (0.3)		–2.2 (0.4)	–1.0 (0.4)	
(95% confidence interval)	(–2.3, –0.9)	(–1.3, 0.1)		(–3.0, –1.5)	(–1.7, –0.2)	
BASFI (0 to 100)	4.0 (0.3)	4.9 (0.2)	0.024	3.5 (0.3)	4.4 (0.3)	0.030
Change from baseline	–1.1 (0.3)	–0.3 (0.2)		–1.7 (0.3)	–0.8 (0.3)	
(95% confidence interval)	(–1.6, –0.6)	(–0.8, 0.2)		(–2.3, –1.1)	(–1.3, –0.2)	
BASMI (0 to 10)	2.3 (0.2)	2.7 (0.2)	0.160	2.2 (0.2)	2.5 (0.2)	0.300
Change from baseline	–0.3 (0.2)	0.1 (0.2)		–0.4 (0.2)	–0.1 (0.2)	
(95% confidence interval)	(–0.7, 0.1)	(–0.3, 0.4)		(–0.8, 0.0)	(–0.5, 0.3)	

Data are adjusted mean (standard error). Analysis of covariance model on change from baseline, with baseline value as a covariate and treatment as a factor. For ASAS-NSAID scores, only patients with nonmissing change from baseline values were included for postbaseline visits. ASAS-NSAID, Assessment of SpondyloArthritis International Society nonsteroidal anti-inflammatory drug use; BASDAI, Bath Ankylosing Spondylitis Disease Activity Index; ASDAS-CRP, Ankylosing Spondylitis Disease Activity Score based on C-reactive protein; PGA, Physician Global Assessment; BASFI, Bath Ankylosing Spondylitis Function Index; BASMI, Bath Ankylosing Spondylitis Metrology Index.

At week 8, 20% and 13% of patients in the etanercept and placebo groups, respectively, achieved ASDAS-CRP inactive disease, whereas 37% and 13% of patients in these groups had ASDAS-CRP moderate disease activity at week 8 (Figure 3B). Normal C-reactive protein levels were observed in 50% and 65% of patients receiving etanercept and placebo at baseline and in 95% and 57% at week 8. Throughout the open-label period, response rates increased for most clinical efficacy endpoints, with steeper increases observed in the placebo/etanercept group than in the etanercept/etanercept group (Additional file 2). At week 16, 54% and 57% of patients treated with etanercept in both the double-blind and open-label periods achieved BASDAI50 and ASAS40 responses, respectively; 49% and 56% of patients who received placebo for 8 weeks followed by etanercept for 8 weeks achieved these responses. As seen with the clinical efficacy endpoints, mean improvements in axSpA signs and symptoms increased further from weeks 8 to 16, with the most pronounced improvements in the placebo/etanercept group.

### Safety

In the double-blind period, treatment-emergent adverse events (AEs) were reported in 81% and 54% of patients in the etanercept and placebo groups, respectively (Table 3). The most common AEs during this period in the etanercept group were rhinitis (12%); asthenia, hypercholesterolemia, injection site hypersensitivity, and injection site reactions (7% each); and headache, injection site erythema, and rash (5%). The most common AEs in the placebo group were asthenia and abdominal pain (6% each); and rhinitis, hypertension, injection site pruritus, alopecia, diarrhea, and pruritus (4% each). A serious AE was reported in one patient in the etanercept group (that is, duodenitis) and in two patients in the placebo group (that is, traffic accident and chest pain).

**Table 3:** **Summary of treatment-emergent adverse events in the etanercept and placebo groups**

	**Double blind (week 8)**		**Open label (week 16)**		**Escape arm**	
**Finding**	**Etanercept 50 mg** **(** ***n*** **= 42)**	**Placebo** **(** ***n*** **= 48)**	**Etanercept 50 mg/etanercept 50 mg** **(** ***n*** **= 31)**	**Placebo/etanercept 50 mg** **(** ***n*** **= 33)**	**Etanercept 50 mg/etanercept 50 mg** **(** ***n*** **= 6)**	**Placebo/etanercept 50 mg** **(** ***n*** **= 11)**
Any adverse event	34 (81.0)	26 (54.2)	12 (38.7)	17 (51.5)	6 (100.0)	9 (81.8)
Serious adverse event	1 (2.4)	2 (4.2)	0	1 (3.0)	0	0
Adverse event leading to discontinuation	4 (9.5)	0	0	2 (6.1)	1 (16.7)	0
Infections	11 (26.2)	10 (20.8)	6 (19.4)	8 (24.2)	2 (33.3)	4 (36.4)
Serious infections	0	0	0	0	0	0

Data presented as number of patients (%).

In the open-label period, AEs were reported in 39% and 52% of patients in the etanercept/etanercept and placebo/etanercept groups, respectively. The most common AEs in the etanercept/etanercept group were headache, injection site pruritus, migraine, and oral herpes (6% each), and in the placebo/etanercept group were injection site erythema (12%), injection site reaction (9%), and headache, nasopharyngitis, and pharyngitis (6% each). One patient in the etanercept/etanercept group had a serious AE (that is, cholecystitis).

AEs of special interest, including infections, were reported in similar proportions of patients in the etanercept and placebo groups. No cases of tuberculosis, demyelinating disorders, malignancies, or deaths were reported. No significant changes from baseline to week 4 or week 8 in diastolic or systolic blood pressure were observed in either the etanercept or placebo group; differences between treatment groups in blood pressure changes were also not significant in the double-blind period. Finally, minimal changes in weight were seen in patients receiving etanercept/etanercept and placebo/etanercept during the double-blind and open-label periods.

## Discussion

Etanercept was associated with NSAID-sparing effects in this prospective, randomized, placebo-controlled study, which was specifically conducted to test this efficacy outcome. The primary outcome measure was recommended by ASAS (for example, ASAS-NSAID score based on NSAID category and daily dose intake) [31]. Importantly, despite the fact that more patients were able to reduce their NSAID intake in the etanercept group, this study also showed the clinically relevant symptomatic effects of etanercept versus placebo.

To our knowledge, this double-blind, placebo-controlled study is the first to evaluate the NSAID-sparing effect of an anti-TNF agent using the ASAS-NSAID score as the primary endpoint. Because of the innovative design of this study, and despite the observed statistically significant difference, estimation of the clinical relevance of the observed results is challenging. In a previous clinical trial in which the ASAS-NSAID score was evaluated as an outcome measure, a change from baseline in ASAS-NSAID score of –24 after open-label anti-TNF therapy was considered to be clinically relevant [43]. The design, in particular the sample size, of the present study was elaborated by defining a between-group difference in ASAS-NSAID score of 30 as clinically relevant. The between-group differences in this score observed in primary and secondary and *post hoc* sensitivity analyses (that is, –27.3, –27.8, –33.3, and –30.4) closely approximate the anticipated difference, suggesting that the study’s statistically significant results are also clinically relevant. Moreover, to further evaluate clinical relevance, *post hoc* analyses of binary endpoints were performed at week 8, including the proportions of patients achieving a 50% reduction in ASAS-NSAID score and very low ASAS-NSAID scores (that is, <10 and 0). Findings of these analyses also support the clinically significant NSAID-sparing effect of etanercept over placebo.

Because patients were advised to decrease their NSAID intake during the treatment period and more patients in the etanercept group were found to have substantially reduced their NSAID intake, a lesser treatment effect of etanercept compared with placebo could reasonably have been expected for conventional outcome measures such as ASAS responses. In fact, in addition to the NSAID-sparing effect of etanercept, this study also demonstrated a symptomatic treatment effect of etanercept over placebo similar to that observed in conventional clinical trials in which NSAID intake was mandatory at baseline and stable NSAID levels were required during the double-blind period. For example, such conventional methodology was followed in the SPINE trial, which assessed the efficacy of etanercept versus placebo in patients with radiographic axSpA who were recruited in similar centers to those participating in the present study [44]. In the SPINE trial, 44% versus 23% of patients receiving etanercept versus placebo, respectively, achieved an ASAS40 response after 12 weeks; whereas in the SPARSE study, 44% versus 21% of patients receiving etanercept versus placebo achieved this endpoint after 8 weeks. Also noteworthy, and consistent with other clinical trials [44,45], the ASDAS demonstrated greater discriminant capacity than the BASDAI in detecting the treatment effect of etanercept in the SPARSE study, as a significant difference was observed between etanercept and placebo at week 8 with the former, but not the latter, measure.

The present study has several noteworthy strengths and weaknesses. The main strength is its design (for example, prospective, randomized, double-blind, placebo-controlled study) with the NSAID-sparing effect specified as the primary objective. The short duration of the study’s double-blind, placebo-controlled period may be considered a weakness. The 8-week duration was selected as it was considered sufficient to demonstrate the NSAID-sparing effect of the biologic agent while limiting the duration of exposure to placebo in patients with this painful, disabling condition. However, the magnitude of such a treatment effect would probably have been greater in a longer trial; as noted in international ASAS recommendations, response rates in patients with axSpA treated with anti-TNF agents have been shown to plateau at and after 12 weeks in phase III clinical trials [46]. Another weakness of the study involved protocol violations related to NSAID intake, which were mainly attributed to investigators’ difficulty in ensuring that enrolled patients had taken NSAIDs for at least 5 days at two-thirds the maximum licensed dosage in the week before the baseline visit. In future studies, investigators may be provided with a calculator or access to an electronic system during screening to improve their ability to check such eligibility criteria. The amount of missing data in patients’ diaries may also be perceived as a weakness. When the study protocol was designed, the optimal means of data collection, either by patient diary or physician interview, was the subject of debate. Given the shortcomings of patient collection using paper diaries encountered in this study (that is, missing data), and the avoidance of such shortcomings in the German Spondyloarthropathy Inception Cohort (GESPIC) [47] and Outcome of Recent Undifferentiated Spondyloarthritis (DESIR) [48] cohort studies, which relied on physician interviews, electronic patient diaries or investigator collection may be considered stronger options in future studies.

Evaluation of the safety profile of etanercept was not the main objective of this study, but no new information was revealed in this area. The study duration was too short to allow evaluation of potential benefits associated with the reduction in NSAID intake in etanercept-treated patients. The observed reductions in NSAID dosage are likely to be more clinically relevant if extended long term; whether such reductions achieved over 8 weeks are clinically relevant has not yet been shown. In particular, no difference was observed in weight or blood pressure changes between the etanercept and placebo groups.

## Conclusions

In this population of patients with axSpA who participated in the SPARSE trial, treatment with etanercept was associated with clinically relevant NSAID-sparing effects, which coincided with significant improvements in conventional clinical outcomes. Additional studies are required to further evaluate the ASAS-NSAID score as a meaningful outcome measure. Long-term observational cohorts are specifically needed to estimate the relationship between NSAID intake and AEs such as renal failure, but other studies are also necessary to determine the optimal means of presenting the obtained results, such as using the ASAS-NSAID score as a continuous or a binary variable.

## Additional files

Additional file 1: Table S1. presenting a summary of missing NSAID diary data at baseline and during the double-blind period. Intention-to-treat population. Additional file 2: Figure S1. showing the proportion of patients in the etanercept/etanercept and placebo/etanercept groups achieving clinical endpoints during the double-blind and open-label periods: **(A)** ASAS partial remission; **(B)** BASDAI50 response; **(C)** ASAS20 response; **(D)** ASAS40 response; and **(E)** PASS. Observed cases.

## Abbreviations

AE: adverse event
ASAS: Assessment of SpondyloArthritis International Society
ASDAS-CRP: Ankylosing Spondylitis Disease Activity Score on the basis of C-reactive protein
axSpA: axial spondyloarthritis
BASDAI: Bath Ankylosing Spondylitis Disease Activity Index
CI: confidence interval
ITT: intent-to-treat
NSAID: nonsteroidal anti-inflammatory drug
PASS: patient acceptable symptom state
SpA: spondyloarthritis
TNF: tumor necrosis factor
