# Supplementary material for: Evaluation of the nonsteroidal anti-inflammatory drug-sparing effect of etanercept in axial spondyloarthritis: results of the multicenter, randomized, double-blind, placebo-controlled SPARSE study
Source: Arthritis Res Ther. 2014 Nov 27;16(6):481. doi: 10.1186/s13075-014-0481-5 (PMC4282738; doi:10.1186/s13075-014-0481-5)
Supplement: Additional file 1: Table S1. — presenting a summary of missing NSAID diary data at baseline and during the double-blind period. Intention-to-treat population. [file 13075_2014_481_MOESM1_ESM.doc]

**Additional file 1: Table S1.** **Summary of missing NSAID diary data at baseline and during the double-blind period**

| **Time point** | **Parameter** | **No. of Patients/Days (%)** | | | |
| --- | --- | --- | --- | --- | --- |
|  |  | **Etanercept 50 mg/ Etanercept 50 mg n = 42** | | **Placebo/ Etanercept 50 mg n = 48** | |
| **Baseline** | Patients with missing diary | 0 | | 1 (2.1) | |
|  | Patients with some missing diary data | 4 (9.5) | | 11 (22.9) | |
|  | Days without information | | | | |
|  | 1 | 1 (2.4) | | 4 (8.3) | |
|  | 2 | 2 (4.8) | | 4 (8.3) | |
|  | 3 | 0 | | 0 | |
|  | 4 | 0 | | 2 (4.2) | |
|  | 5 | 0 | | 0 | |
|  | 6 | 1 (2.4) | | 1 (2.1) | |
| **Week 4** | Patients with missing diary | | 7 (16.7) | 5 (10.4) | |
|  | Patients with some missing diary data | | 3 (7.1) | 9 (18.8) | |
|  | Days without information | | | | |
|  | 1 | | 3 (7.1) | 2 (4.2) | |
|  | 2 | | 0 | 2 (4.2) | |
|  | 3 | | 0 | 2 (4.2) | |
|  | 4 | | 0 | 1 (2.1) | |
|  | 5 | | 0 | 2 (4.2) | |
|  | 6 | | 0 | 0 | |
| **Week 8** | Patients with missing diary | | 5 (11.9) | 4 (8.3) | |
|  | Patients with some missing diary data | | 3 (7.1) | 7 (14.6) | |
|  | Days without information | | | | |
|  | 1 | | 0 | | 2 (4.2) |
|  | 2 | | 0 | | 1 (2.1) |
|  | 3 | | 1 (2.4) | | 1 (2.1) |
|  | 4 | | 0 | | 2 (4.2) |
|  | 5 | | 0 | | 0 |
|  | 6 | | 2 (4.8) | | 1 (2.1) |

Intention-to-treat population.
