# Supplementary figures and images for: Evaluation of the nonsteroidal anti-inflammatory drug-sparing effect of etanercept in axial spondyloarthritis: results of the multicenter, randomized, double-blind, placebo-controlled SPARSE study
Source: Arthritis Res Ther. 2014 Nov 27;16(6):481. doi: 10.1186/s13075-014-0481-5 (PMC4282738; doi:10.1186/s13075-014-0481-5)

**A**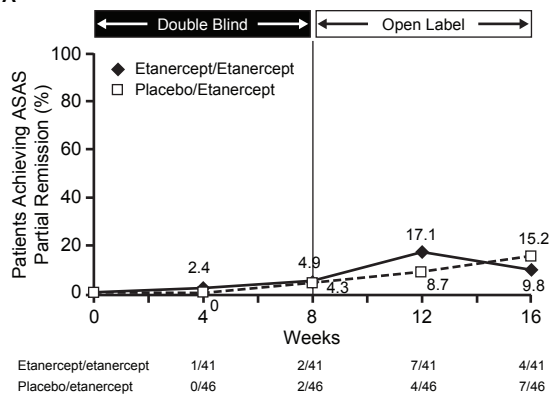**B**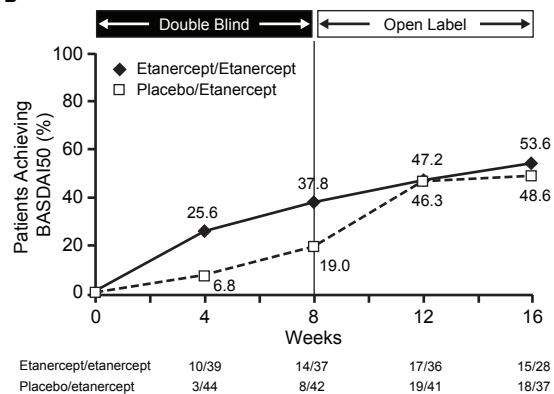**C**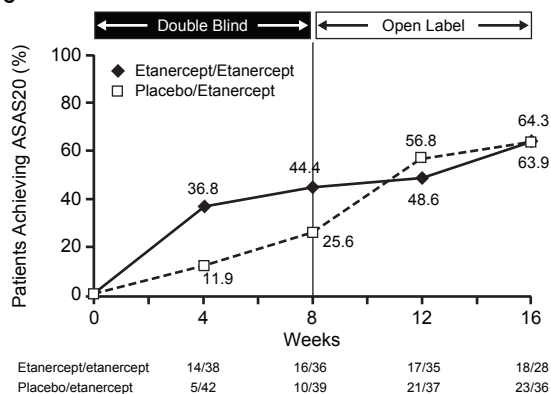**D**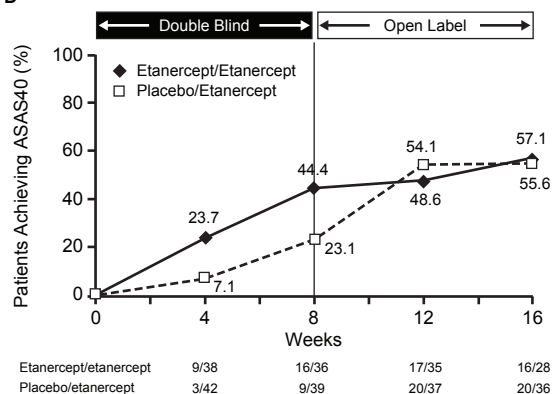**E**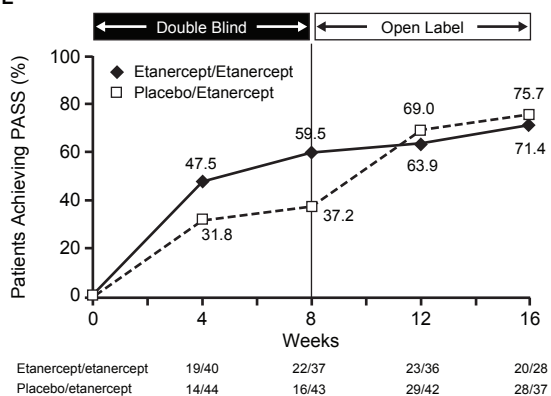

Supplement: Additional file 2: Figure S1. — showing the proportion of patients in the etanercept/etanercept and placebo/etanercept groups achieving clinical endpoints during the double-blind and open-label periods: (A) ASAS partial remission; (B) BASDAI50 response; (C) ASAS20 response; (D) ASAS40 response; and (E) PASS. Observed cases. [file 13075_2014_481_MOESM2_ESM.pdf]
